# Potential oral probiotic Lactobacillus pentosus MJM60383 inhibits Streptococcus mutans biofilm formation by inhibiting sucrose decomposition

**DOI:** 10.1080/20002297.2022.2161179

**Published:** 2022-12-28

**Authors:** Mingkun Gu, Joo-Hyung Cho, Joo-Won Suh, Jinhua Cheng

**Affiliations:** a Interdisciplinary Program of Biomodulation, Myongji University, Yongin, Republic of Korea; b Myongji Bioefficacy Research Center, Myongji University, Yongin, Republic of Korea

**Keywords:** *Lactobacillus pentosus*, oral probiotics, biofilm, *streptococcus mutans*, sucrose decomposition

## Abstract

*Streptococcus mutans* is known as a contributor to dental caries. In this work, *Lactobacillus pentosus* MJM60383 was selected for its strong antagonistic activity against *S. mutans* and was characterized by good oral probiotic properties including lysozyme tolerance, adhesive ability to oral cells, good aggregation (auto-aggregation, co-aggregation) ability, hydrogen peroxide production and inhibition of biofilm formation of *S. mutans*. *L. pentosus* MJM60383 also exhibited safety as a probiotic characterized by no hemolytic activity, no D-lactate production, no biogenic amine production, and susceptibility to antibiotics. Furthermore, the biofilm formation of *S. mutans* was also significantly inhibited by the supernatant of *L. pentosus* MJM60383. An anti-biofilm mechanism study revealed that sucrose decomposition and the production of water-insoluble exopolysaccharides by *S. mutans* were inhibited by the treatment with *L. pentosus* MJM60383 supernatant. Real-time PCR analysis indicated that the supernatant of *L. pentosus* MJM60383 significantly inhibited the mRNA expression of *S. mutans* glycosyltransferases, which synthesize glucan to construct biofilm architecture and mediate bacterial adherence. Our study demonstrated *L. pentosus* MJM60383 as a potential oral probiotic and revealed its anti-biofilm mechanism.

## Introduction

*Streptococcus mutans* is considered to be the main causative agent of human dental caries, and one of its important virulence properties is the ability to form a biofilm known as dental plaque on the tooth surface [1]. *S. mutans* produces acid in the biofilm by fermentation of dietary carbohydrates such as sucrose, and the acid can demineralize the tooth surface, leading to dental caries. Adherence of *S. mutans* to dental surfaces is the first step in the formation of biofilms which are mediated by sucrose-dependent and sucrose-independent mechanisms [2,3].

Probiotics are live microorganisms that offer beneficial effects to the host [4]. According to the Food and Agriculture Organization/World Health Organization guidelines, probiotics should be safe to use. Safety assessments commonly include no hemolytic activity, no D-lactate production, no biogenetic amine production, and no transferrable antibiotic resistance [5]. Most probiotics are classified as the genera *Lactobacillu*s or *Bifidobacterium* [6]. In particular, *Lactobacillus* species make up a prominent part of oral microbiota to interfere with pathogenicity [7,8]. An effective oral probiotic has been screened so far by evaluating its efficacy *in vitro*, including producing antimicrobial components (hydrogen peroxide, lactic acid, bacteriocins) that can inhibit the growth of oral pathogens, high tolerance to the environmental stress (acid and lysozyme tolerance) [9], no propagating antibiotic resistance, good auto-aggregation and co-aggregation ability [10] and adhesion to oral epithelial cells [11].

Recent studies have revealed the potential of lactic acid bacteria (LAB) in oral health [12]. For example, *Lactobacillus acidophilus* supernatants were reported to ameliorate periodontitis and gingivitis [13]. *Lactobacillus reuteri*, *Lactobacillus rhamnosus* GG (LGG), and *Lactobacillus plantarum* inhibit the biofilm formation of *S. mutans* [14]. *Weissella cibaria* culture supernatants exhibited antibacterial activity against oral pathogens. The impacts of the supernatant were dependent on the hydrogen peroxide or organic acid produced by *W. cibaria* [15]. Another study showed that the culture supernatants of *Lactobacillus kefranofaciens* have anti-biofilm and antimicrobial activities against oral pathogens [16]. However, studies on oral probiotics are still limited compared to gut probiotics.

This study aimed to characterize *L. pentosus* MJM60383 as a potential oral probiotic. Thus, the oral probiotic properties and safety of *L. pentosus* MJM60383 were investigated. In addition, the anti-biofilm activity was also evaluated. Furthermore, the anti-biofilm mechanism was elucidated from the aspect of biofilm structure alteration, water-insoluble EPS synthesis, sucrose decomposition, and expression of biofilm-related genes.

## Materials and methods

### Microorganisms and growth conditions

*Streptococcus mutans* KCTC 3065 was obtained from the Korean Collection for Type Culture (Daejeon, Republic of Korea) and was grown on Brain Heart Infusion (BHI) media at 37°C for 24 h. *Lactobacillus pentosus* MJM60383 was isolated from fermented vegetables in our laboratory. LGG was obtained from American Type Culture Collection. LGG was reported to have good effects on oral cavities by inhibiting the growth of *S. mutans* and reducing the risk of dental caries. Thus, LGG was used as a control strain in this study. Both LGG and *L. pentosus* MJM60383 were cultivated in de Man-Rogosa-Sharpe (MRS) media at 37°C for 24 h.

### In vitro safety assessment

To assess whether *L. pentosus* MJM60383 is safe to be a probiotic, we tested its hemolytic activity, D-lactic acid production, biogenic amine production, and antibiotic susceptibility.

The hemolytic activity of *L. pentosus* MJM60383 and LGG were assessed using MRS agar plates added 5% defibrinated sheep blood. *L. pentosus* MJM60383 was streaked on plates and incubated at 37°C for 24 h. Hemolytic activity was observed with transmitted light and determined by the photochromic properties around the colonies.

For the evaluation of D-lactate production, *L. pentosus* MJM60383 and LGG were grown in MRS broth at 37°C for 24 h. The supernatant of *L. pentosus* MJM60383 and LGG were analyzed using a D-lactic acid assay kit (Megazyme, K-DATE) according to the manufacturer’s protocol.

For assessment of biogenic amine produced by *L. pentosus* MJM60383, the decarboxylase agar plates (%) (tryptone, 0.02 g; manganese sulfate, 0.5 g; yeast extract, 0.5 g; sodium chloride, 0.25 g; glucose, 0.05 g; tween 80 g, 0.005 g; bromocresol purple, 0.1 g; magnesium sulfate, 0.005 g; ferrous sulfate, 0.2 g; thiamine, 0.001 g; dipotassium phosphate, 0.5 g; meat extract, 0.2 g; calcium carbonate, 0.004 g; ammonium citrate, 0.01 g; pyridoxal 5-phosphate, 0.006 g; agar, 20 g; distilled water, 1000 mL; pH, 5.3) were prepared by adding different amino acids (L-phenylalanine, L-histidine, L-lysine, L-tyrosine, and L-ornithine) [17]. The decarboxylase medium without amino acids supplementation served as a control. *L. pentosus* MJM60383 and LGG were streaked on plates and incubated at 37 ºC for 48 h. After incubation, biogenic amine production was determined by the appearance of purple color around the colony.

For the antibiotic susceptibility assay, the minimal inhibitory concentrations (MICs) of *L. pentosus* MJM60383 and LGG were assessed using a serial two-fold dilution method in MRS broth according to the guidance of the European Food Safety Authority (EFSA) [18]. Briefly, nine common antibiotics were dissolved in the MRS broth at a concentration of 256 µg/mL, and serially two-fold diluted into 96-well plates. An equal volume of *L. pentosus* MJM60383 or LGG strain suspension (McFarland 0.5) was added to each of the wells and incubated for 24 h at 37°C, respectively. After incubation, the MIC is determined as the lowest concentration of the antibiotics that inhibits the growth of *Lactobacillus*. All the safety assessment experiments were done in triplicate.

### Antagonistic activity

The antagonistic activity was determined by agar diffusion assay [19]. Briefly, overnight culture of *S. mutans* was diluted with MRS broth to OD600 = 1 (~1 × 10^8^ CFU/mL). The *S. mutans* suspension was mixed with BHI soft agar (0.7% agar) at a ratio of 0.1% (v/v). Then 10 ml of the soft agar was poured onto an MRS agar (1.5% agar) plate to make a top-covered assay plate. A small hole was made on the top-covered assay plate, and 5 µL of an overnight culture of *L. pentosus* MJM60383 was loaded into the hole. LGG was used as a control. Plates were incubated for 48 h at 37°C. Inhibition zones were measured after incubation. This experiment was done in triplicate.

### Lysozyme tolerance of isolates

The effect of lysozyme on the growth of *L. pentosus* MJM60383 was tested [9]. The overnight culture of *L. pentosus* MJM60383 (McFarland 0.5) was seeded in MRS broth (0.7% soft agar) and poured onto plates. Then an Oxford cup was placed on agar plates, and 25 μL lysozyme solution (1 mg/mL) was loaded into each well. The plates were incubated for 24 h at 37°C to observe inhibition zones. This experiment was performed in triplicate.

### Adhesion to oral epithelial cell

The YD-38 (oral epithelial carcinoma cell line) was used to test the adhesive capability of *L. pentosus* MJM60383 and LGG [20]. YD-38 cells (purchased from Korean Cell Line Bank) were grown in RPMI 1640 medium (with L-glutamine 300 mg/L, 25 mM HEPES, and 25 mM NaHCO_3_) supplemented with 10% (v/v) fetal bovine serum and 100 µg/mL of streptomycin and 100 IU/mL of penicillin at 37°C under 5% CO_2_. The YD-38 cells (3.0 × 10^5^) were seeded in 6-well plates to reach a complete confluent monolayer and washed with PBS. *L. pentosus* MJM60383 cells were suspended in RPMI 1640 medium at a concentration of 10^8^ CFU/mL and then treated with YD-38 cells. Cells and bacteria were incubated at 37°C under 5% CO_2_ for 2 h. After incubation, the plates were washed 3 times with RPMI 1640 medium to remove non-adhering bacteria.

For quantification of the adhesive ability of *L. pentosus* MJM60383 and LGG to YD-38 cells, cells and bacteria were detached from the plate by treatment with 0.25% trypsin-EDTA (Gibco, Canada), then lysed in PBS. This solution was diluted and plated on an MRS agar plate. Colony-forming units of *L. pentosus* MJM60383 were counted after 48 h. This experiment was performed in triplicate. Adhesive capacity was determined as:

Adhesive capacity (%) = CFU of adherent bacteria/CFU of inoculated bacteria

### Analysis of acid production

The pH values of the overnight culture of LAB were measured. The content of lactic acid produced by *L. pentosus* MJM60383 and LGG was measured by high-performance liquid chromatography (HPLC) [21]. The overnight culture of LAB was centrifuged at 4,000 rpm for 10 min, and the supernatant was filtered through a 0.22 μm membrane filter (Sartorius, Germany). The HPLC analysis was performed with a ZORBAX SB-C18 column (150 mm × 4.6 mm, 5 μm, Agilent, USA) connected to a binary HPLC pump (Waters 1525, USA), and eluted with sodium dihydrogen phosphate buffer (pH 2.5) at a flow rate of 1 mL/min. The lactic acid was detected by a photodiode array detector (Waters, USA) at 210 nm, and the injection volume is 20 μL. This experiment was repeated in triplicate.

### Hydrogen peroxide production

Both *L. pentosus* MJM60383 and LGG were grown in MRS broth for 24 h at 37°C. The cell-free supernatant was collected by centrifugation at 4,000 rpm for 10 min. Hydrogen peroxide in the supernatant was measured using semiquantitative test strips (Merck, Poole, UK) according to the manufacturer’s protocol [10]. This experiment was performed in triplicate.

### Auto-aggregation and co-aggregation assay

The auto-aggregation and co-aggregation ability of *L. pentosus* MJM60383 was tested according to the method described before [22]. *L. pentosus* and LGG were incubated in MRS broth for 24 h at 37°C. The cell pellet of *L. pentosus* and LGG were harvested by centrifugation at 7,200 rpm for 15 min and washed three times with PBS, then resuspended in PBS (10^9^ CFU mL^−1^). Each bacterial suspension was vortexed for 10 s and statically incubated for 4, 20, and 24 h. One mL of the upper part of each sample solution was taken at a different time interval, and the absorbance at 600 nm was determined by a spectrophotometer (Optizen, Daejeon, Republic of Korea). The percentage of auto-aggregation was calculated as follows:

Auto-aggregation (%) = [1− (A_t_/A_0_)×100]

Where A_t_ represents the absorbance at time intervals of 4, 20, and 24 h, and A_0_ indicates the absorbance at time 0.

For the co-aggregation assay, LAB suspensions were prepared (10^9^ CFU mL^−1^) as described above. *S. mutans* was grown in BHI broth for 24 h at 37°C. Then, the cells of *S. mutans* were harvested by centrifugation at 7,000 rpm for 15 min and resuspended in PBS at a concentration of 10^9^ CFU mL^−1^. *S. mutans* (SM, 2 mL) suspension was mixed with 2 ml of the LAB suspensions. Samples containing 4 mL of each bacterial suspension were used as a control. Each suspension was vortexed for 10 s and incubated statically at room temperature. Then 1 ml of the upper part of each suspension was measured at an absorbance of 600 nm at time intervals of 4, 20, and 24 h by a spectrophotometer. The co-aggregation percentage was determined as follows:

**Table:** 

Co-aggregation (%) = [(A_SM_ + A_LAB_)/2 − (A_mix_)]/(A_SM_ + A_LAB_)/2 × 100	

Where A_SM_ and A_LAB_ are the optical density (OD) of *S. mutans* and LAB strains, respectively. A_mix_ represents the OD of the mixture of *S. mutans* and LAB. All the experiments were performed in triplicates.

### Effect of L. pentosus MJM60383 on the biofilm formation in a co-culture model

Biofilms were determined using safranin red assay [23]. For the co-culture model, both the overnight *S. mutans* and LAB culture were adjusted to OD600 of 1 and then diluted 100-fold with BHI containing 1% sucrose. For the treatment group, 20 µL of *S. mutans* and 20 µL of live LAB culture or heat-killed LAB culture were added to 160 µL of BHI medium containing 1% sucrose and incubated for 24 h at 37°C. After incubation. The biofilms in the 96-well plates were washed three times with distilled water and air dried. The biofilms were stained with 0.2% safranin red for 35 min at room temperature. After incubation, the dye was decanted from the wells and the biofilms were washed three times with distilled water without agitation, and then 125 μL of 33% acetic acid was added to extract the dye. The plates were shaken for 30 s before quantification of absorbance at 492 nm by a microtiter plate reader (Tecan, infinite M200PRO, Austria). For the control group, 20 µL of *S. mutans* and 20 µL of MRS broth were added to 160 µL of BHI medium containing 1% sucrose and incubated for 24 h at 37°C. This experiment was performed in triplicate.

### Scanning electron microscopy

A cover glass (1 × 1 cm) was sterilized and placed in the well of a 6-well plate. As described above, for the LAB treatment group, 20 uL of *S. mutans* (OD 600 = 0.01), 20 µL of LAB culture (OD 600 = 0.01) and 160 µL of BHI medium (containing 1% sucrose) were added to the wells containing the cover glass, and the 6-well plates were anaerobically incubated at 37°C for 24 h. For the control group, 20 µL of MRS broth was added instead of LAB culture.

For scanning electron microscopic (SEM) observation [24], the cover glass was gently washed with PBS once, fixed with PBS containing 4% paraformaldehyde and 2.5% glutaraldehyde, dehydrated in graded ethanol solutions, and finally sputter-coated with gold for the SEM observation. This experiment was performed in triplicate.

### Effect of L. pentosus MJM60383 supernatant on the biofilm formation

For the preparation of the supernatant, *L. pentosus* MJM60383 was incubated in 15 mL MRS broth at 37°C for 24 h. The culture broth was centrifuged, and the supernatant was filtered through a 0.22 µm membrane filter. To remove the influence of lactic acid, bacteriocin, and hydrogen peroxide, the supernatant of *L. pentosus* MJM60383 was adjusted to pH 6.8 with 10 N NaOH, or treated with 1 mg/mL trypsin, or treated with 0.5 mg/mL catalase. To determine the effect of LAB supernatant on *S. mutans* biofilm formation, *S. mutans* was prepared as described above. Then 20 µL *S. mutans* and 20 µL LAB supernatant, and 160 µL BHI medium supplemented with 1% sucrose were added to 96-well plates and incubated at 37°C for 24 h. For the control group, 20 µL of MRS medium was added instead of LAB supernatant. Biofilm formation by *S. mutans* was determined as described above. This experiment was performed in triplicate.

### The anti-adherence activity of L. pentosus MJM60383 supernatant

The anti-adherence assay was conducted according to the method of Islam *et al.* [25] with slight modifications. The 20 μL of *S. mutans* (OD _600_ = 0.01) was treated with 20 μL of *L. pentosus* MJM60383 supernatant in 96-well plates containing 160 µL of BHI with 1% sucrose, and the plates were incubated for 24 h at 37°C. In the control group, MRS medium was used instead of the supernatant of *L. pentosus* MJM60383. After incubation, planktonic cells were removed from the plate and the bacteria density was measured at OD600. The adherent cells were washed three times with phosphate saline buffer (PBS) and removed from the plate by 0.5 N NaOH. Bacterial density in the biofilm was quantified at OD 600 nm. This experiment was performed in triplicate. The percentage of adhesive *S. mutans* was calculated below:

Percentage adherence = (OD_600_ of biofilm cells/(OD_600_ of biofilm cells + OD_600_ of planktonic cells)) × 100.

### Determination of sucrose content in the medium treated with L. pentosus MJM60383 supernatant

Twenty microliters of *S. mutans* culture (OD600 = 0.01), 20 µL of *L. pentosus* MJM60383 supernatant, and 160 μL of BHI medium containing 1% sucrose were added to the wells of a 96-well plate and incubated at 37°C for 24 h. The experiment treated with MRS broth instead of *L. pentosus* MJM60383 supernatant served as a control. The sucrose content in the supernatant was assessed by HPLC with a refractive index detector (Agilent Technologies, Boeblingen, Germany) [26]. All samples were conducted on the Aminex HPX-87P column and eluted with distilled water as the mobile phase at a flow rate of 1 mL/min for 30 min. The injection volume was 30 μL. This experiment was performed in triplicate.

### Water-insoluble EPS measurement

The water-insoluble EPS was determined by using the anthrone method [27]. Briefly, *S. mutans* was treated with PBS or *L. pentosus* MJM60383 supernatant and incubated in 12-well plates containing BHI medium with 1% sucrose for 24 h at 37°C. After incubation, the supernatant was removed from the well, biofilm was washed 3 times with sterile water, fully scraped by a spatula, and collected by centrifugation. Then the sediment was resuspended in NaOH (0.4 M) solution. After centrifuging at 7000 rpm for 10 min, the supernatant of samples was treated with anthrone reagent (v:v, 1:3) at 90°C for 17 min. The absorbance of the mixture was measured at 625 nm, and the content of water-insoluble EPS produced by *S. mutans* was calculated using glucose standard curves [28]. This experiment was performed in triplicate.

### Effect of L. pentosus MJM60383 supernatant on the expression of the biofilm-related genes

The overnight culture of *S. mutans* (adjusted to OD_600_ = 0.01) was treated with *L. pentosus* supernatant and cultured in BHI medium supplemented with 1% sucrose at 37°C for 6 or 24 h. After incubation, biofilm cells were collected by centrifugation at 4,000 rpm for 10 min. The total RNA of biofilm cells was extracted using Trizol™ reagent (Invitrogen, CA, USA) according to the method as previously described [29]. cDNA was synthesized using the PrimeScript™ RT reagent Kit with gDNA Eraser (Takara, Shiga, Japan). The effect of *L. pentosus* MJM60383 supernatant on the expression of genes was assessed by real-time quantitative PCR conducted on LightCycler® 96 System (Roche, Basle, Switzerland). The primers used in this study were listed in Table 1. The reaction mixtures (20 µL) contained 10 μL of Real Universal PreMix with SYBR Green I (Takara, Shiga, Japan), 1 μL of template cDNA, 1 μL of each pair of primers, and 8 μL of distilled water. A 35-cycle thermal reaction was performed, including denaturation at 94°C for 10 min, annealing at 56°C for 10 s, and extension at 72°C for 20 s. 16S rRNA, the housekeeping gene was used as a control. The relative gene expression was calculated following the 2^−^^△^^△^^CT^ analysis method [30]. This experiment was performed in triplicate.

**Table 1.** Sequences of primers used for PCR and RT-PCR.

Gene*	Primer sequence (5’-3’)		Ta** (ºC)	Amplicon size (bp)
	Forward	Reverse		
*ftf*	AAATATGAAGGCGGCTACAACG	CTTCACCAGTCTTAGCATCCTGAA	56	101
*gtfb*	AGCAATGCAGCCAATCTACAAAT	ACGAACTTTGCCGTTATTGTCA	55	96
*gtfc*	GGTTTAACGTCAAAATTAGCTGTATTAGC	CTCAACCAACCGCCACTGTT	54	91
*gtfd*	TGTCTTGGTGGCCAGATAAAC	GAACGGTTTGTGCAGCAAGG	62	132
*brpA*	CGTGAGGTCATCAGCAAGGTC	CGCTGTACCCCAAAAGTTTAGG	59	148
*gbpB*	CGTGTTTCGGCTATTCGTGAAG	TGCTGCTTGATTTTCTTGTTGC	58	108
*comDE*	ACAATTCCTTGAGTTCCATCCAAG	TGGTCTGCTGCCTGTTGC	55	81
*spaP*	GACTTTGGTAATGGTTATGCATCAA	TTTGTATCAGCCGGATCAAGTG	53	121
*aguD*	ATCCCGTGAGTGATAGTATTTG	CAAGCCACCAACAAGTAAGG	62	80
*atpD*	CGTGCTCTCTCGCCTGAAATAG	ACTCACGATAACGCTGCAAGAC	52	101
*16S rRNA*	CCTACGGGAGGCAGCAGTAG	CAACAGAGCTTTACGATCCGAAA	57	247

*ftf: encoding levansucrase enzyme (fructosyltransferase); gtfb, encoding glucosyltransferase I; gtfc, glucosyltransferase SI; gtfd, glucosyltransferase S; brpA, Biofilm-regulation protein, gbpB, glucan-binding protein; comDE, Competence-stimulating peptide; spaP, Cell surface antigen; aguD, Agmatine: putrescine antiporter; atpD, F-ATPase beta-subunit; 16S rRNA, 16S ribosomal RNA gene.

† 16s rRNA gene was used as an internal control.

**Ta: annealing temperature,

### Statistical analysis

Data were analyzed using GraphPad Prism version 8.1.0. Values are presented as mean ± SD. Statistical significance was determined by a two-tailed unpaired Student’s t-test between two groups, the comparison of more than 2 groups was evaluated with one-way ANOVA, followed by Dunnett’s multiple comparison test. A p-value of <0.05 was considered statistically significant.

## Results

### Safety assessment of L. pentosus MJM60383

A probiotic must be non-toxic and safe. As shown in Table 2, Strain *L. pentosus* MJM60383 showed no hemolytic activity, no D-lactate production, and no biogenic amine production as confirmed by observation. Strain *L. pentosus* MJM60383 also showed susceptibility to chloramphenicol, ampicillin, gentamycin, streptomycin, erythromycin, and clindamycin according to the cutoff value recommended by EFSA.

**Table 2.** Safety assessment of *L. pentosus* MJM60383.

Safety assessment	*L. pentosus* MJM60383	LGG
Hemolytic activity	-	-
D-lactate production	-	-
Biogenic amine production		
L-Phenylalanine	-	-
L-Lysine	-	-
L-Ornithine	-	-
L-Histidine	-	-
L-Tyrosine	-	-
Antibiotic susceptibility (MIC, µg/ml)		
Chloramphenicol	4 (S)	4 (S)
Ampicillin	0.25 (S)	4 (S)
Tetracycline	16 (R)	2 (S)
Vancomycin	256 (n.r.)	256 (n.r)
Gentamycin	4 (S)	16 (R)
Kanamycin	64 (R)	64 (R)
Streptomycin	64 (S)	128 (R)
Erythromycin	0.5 (S)	0.5 (S)
Clindamycin	0.5 (S)	0.5 (S)

-, No activity.

†R, resistant; S, sensitive; n.r., not required.

### Oral probiotics properties of L. pentosus MJM60383

The oral probiotic properties of *L. pentosus* MJM60383 were evaluated (Table 3). Antagonistic activity is a very important trait of probiotics. *L. pentosus* MJM60383 showed strong antagonistic activity against *S. mutans*, the main pathogen for dental caries. Moreover, *L. pentosus* MJM60383 showed higher activity than LGG which was used as a reference probiotic strain.

**Table 3.** Oral probiotic properties of *L. pentosus* MJM60383.

Oral probiotics properties	*L. pentosus* MJM60383	LGG
Antagonistic activity (Inhibition zone, mm)	30 ± 0.03	27 ± 0.04
Lysozyme tolerance (1 mg/mL)	T*	T*
Adhesion to YD-38 cell (%)	20 ± 0.02	18 ± 0.05
Lactic acid (g/L)	13.89 ± 0.12	14.0 5 ± 0.13
Hydrogen peroxide production (mM)	0.06–0.14	<0.015
Auto-aggregation (%)		
4 h	19.75	16.98
20 h	47.97	46.19
24 h	50.30	47.75
Co-aggregation (%)		
4 h	14.25	13.56
20 h	27.50	26.95
24 h	38.35	35.13

*T, tolerant

Each experiment was statistically analyzed.

An oral probiotic should survive in the oral cavity and colonize the oral tissues. Lysozyme is an antimicrobial enzyme naturally found in saliva that leads to cell death by cleaving the peptidoglycan component of bacterial cell walls. *L. pentosus* MJM60383 and LGG showed tolerance to lysozyme at a concentration of 1.0 mg/mL. The adhesive ability of *L. pentosus* MJM60383 and LGG to the oral epithelial cell monolayer was 20% and 18%, respectively (Table 3).

Organic acid (mostly lactic acid) and H_2_O_2_ are important metabolites that are produced by nearly all lactic acid bacteria and contributed to the inhibition of oral pathogens. The pH values for the culture broth of *L. pentosus* MJM60383 and LGG were determined as 3.7 and 3.8, respectively. The concentrations of lactic acid produced by *L. pentosus* MJM60383 and LGG were 13.89 ± 0.12 and 14.05 ± 0.13 g/L, respectively. *L. pentosus* MJM60383 produced hydrogen peroxide in the range of 0.06 − 0.14 mM, which was higher than LGG (<0.015 mM) (Table 3).

As shown in Table 3, *L. pentosus* MJM60383 showed a good ability of aggregation (auto-, co-aggregation). The auto-aggregation and coaggregation ability of *L. pentosus* MJM60383 were higher than LGG at the indicated time point.

### Effect of L. pentosus MJM60383 on S. mutans biofilm formation

**Figure 1.**
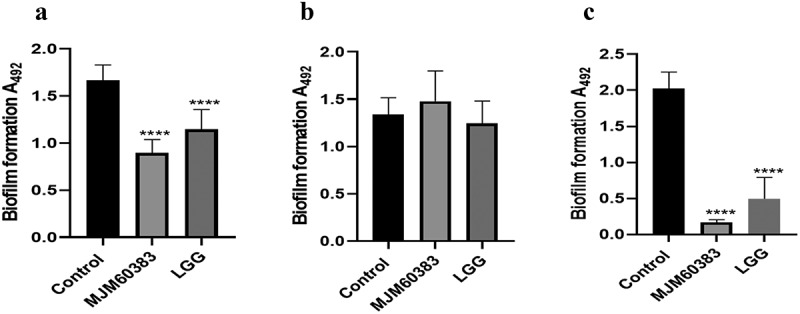
**Effect of LAB on *S. mutans* biofilm formation in a co-culture model**. The overnight culture of *S. mutans* was diluted 100-fold with BHI supplemented with 1% sucrose before treatment with LAB. (A) *S. mutans* was co-cultured with live LAB. (B) *S. mutans* was co-cultured with heat-killed LAB. (C) *S. mutans* was treated with LAB supernatant. In the control group, MRS broth was treated instead of LAB supernatant. Asterisks indicate a significant difference analyzed using the one-way ANOVA with Dunnett’s multiple comparison test. ****p <0.0001, significantly different from the control group.

Both live *L. pentosus* MJM60383 and LGG significantly suppressed *S. mutans* biofilm formation. Compared to the control group, biofilm formation was inhibited 33% and 14% by *L. pentosus* MJM60383 and LGG, respectively, in the co-culture model (Figure 1A). Moreover, *L. pentosus* MJM60383 showed higher anti-biofilm activity than LGG. However, heat-killed *L. pentosus* MJM60383 and LGG did not inhibit the biofilm formation of *S. mutans* (Figure 1B). In addition, we also investigated the effect of the supernatant of *L. pentosus* MJM60383 and LGG on *S. mutans* biofilm formation. The supernatant of *L. pentosus* MJM60383 and LGG dramatically suppressed biofilm formation. *L. pentosus* MJM60383 supernatant showed higher activity than LGG supernatant. The reduction percentage was 91.5% (Figure 1C).

**Figure 2.**
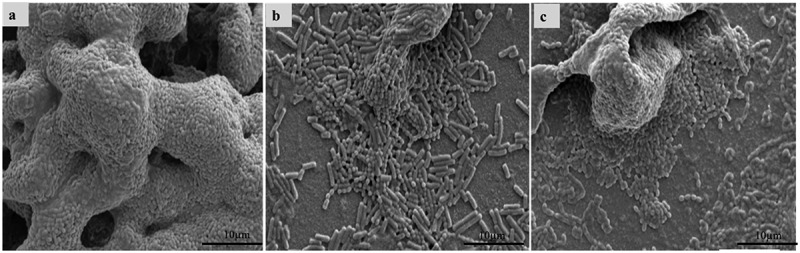
**Scanning electron microscopy (SEM) observation**. Structures of biofilm. (a) Biofilm structure of *S. mutans* only. The biofilm structure of *S. mutans* when co-cultured with live *L. pentosus* MJM 60383 (b) or LGG (c). The structure of biofilm was observed by SEM at 3000× magnification.

As shown in Figure 2, SEM observation showed that *S. mutans* formed a thick and compact biofilm structure on the surface of the glass (Figure 2a). When *S. mutans* was co-cultured with live LAB, the structures of biofilm were thinner and looser compared to the control group. Moreover, fewer bacteria were observed in the *L. pentosus* MJM60383-treated group than in the LGG-treated group.

### L. *pentosus MJM60383 supernatant inhibits biofilm formation, adhesion, and water-insoluble EPS synthesis of S. mutans*

**Figure 3.**
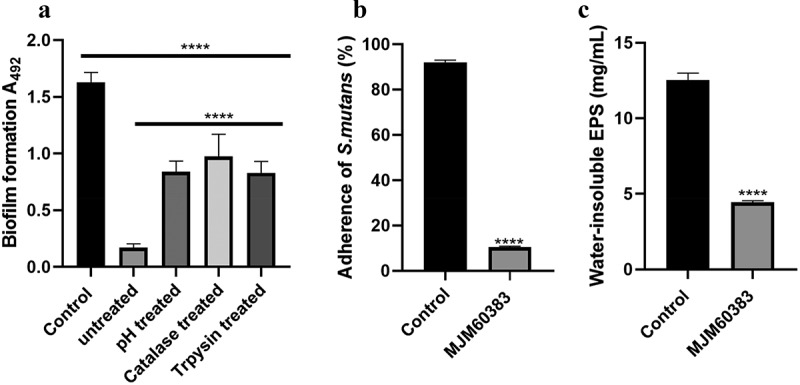
**Effect of *L. pentosus* MJM60383 supernatant on *S. mutans* biofilm formation**. (A) Biofilm formation by *S. mutans* in the presence of treated and untreated *L. pentosus* MJM60383 supernatant. Control was treated with MRS media instead of *L. pentosus* MJM60383 supernatant. (B) Cell adherence of *S. mutans* in the presence of *L. pentosus* MJM60383 supernatant. In control wells, the supernatant was replaced by sterile MRS broth. (C) the amount of water-insoluble EPS in the *S. mutans* biofilms. Quantitative data of the water-insoluble EPS amount of *S. mutans* biofilms measured by the anthrone method. Data are presented as means ± standard deviations. Asterisks indicate a significant difference analyzed using the one-way ANOVA with Dunnett’s multiple comparison test. ****p <0.0001, significantly different from the control.

To investigate the effective factors in the supernatant, *L. pentosus* MJM60383 supernatant was neutralized, or treated with trypsin or catalase to remove the activity caused by lactic acid, bacteriocins, and H_2_O_2_. The biofilm was reduced by 41.5% when treated with neutralized supernatant. Catalase-treated and trypsin-treated supernatant also significantly inhibited 40.1% and 49.1% biofilm formation, respectively (Figure 3A).

Adhesion of *S. mutans* to the well surface was strongly inhibited by treatment with *L. pentosus* MJM60383 supernatant. In the control group, 91% of the bacteria were embedded in the biofilm, while only 10% of the bacteria were embedded in the biofilm in the *L. pentosus* MJM60383 supernatant-treated group (Figure 3B).

In addition, the production of water-insoluble EPS was significantly inhibited by treatment with *L. pentosus* MJM60383 supernatant. The water-insoluble EPS produced by *S. mutans* in the control group was 12.76 ± 0.12 mg/mL, while it was 4.48 ± 0.13 mg/mL in the supernatant-treated group (Figure 3C).

**Figure 4.**
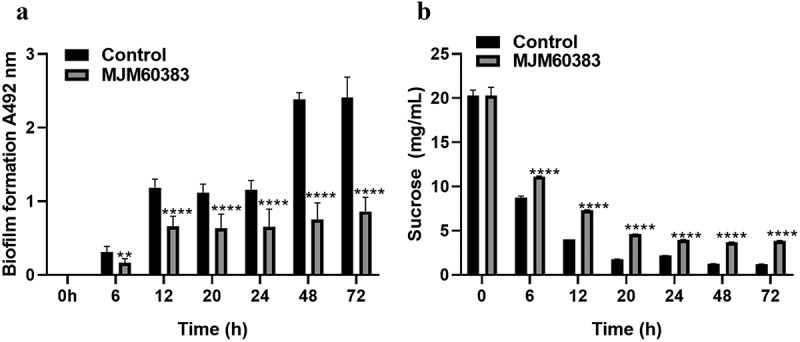
***L. pentosus*** **MJM60383 supernatant inhibits biofilm formation from the adherent stage and sucrose decomposition of *S. mutans***. (A) *S. mutans* was grown on 96-well plates at 37°C for 6, 12, 20, 24, 48, and 72 h in the presence or absence of supernatant. Biofilm formation - was determined by the safranine dye. (B) *L. pentosus* MJM60383 supernatant inhibit sucrose decomposition. *S. mutans* was grown in a BHI medium supplemented with 1% sucrose at 37°C for 0, 6, 12, 20, 24, 48, and 72 h in the presence or absence of *L. pentosus MJM60383* supernatant. The culture supernatants were subjected to HPLC-RID for sucrose detection. Data represent the mean and standard deviation. Asterisks indicate a significant difference analyzed using the one-way ANOVA with Dunnett’s multiple comparison test. **p <0.01, ****p <0.0001.

- ***L. pentosus MJM60383 supernatant inhibits sucrose decomposition of S. mutans***
- *S. mutans* decompose sucrose in the medium to form a biofilm. After treatment with *L. pentosus* MJM60383 supernatant, the biofilm formation of *S. mutans* was significantly inhibited during the incubation time (Figure 4A), and the sucrose decomposition was also reduced (Figure 4B). In the control group (without treatment with supernatant), the concentration of sucrose in the BHI medium was decreased from 20 mg/mL (at 0 h) to 1.2 ± 0.02 mg/mL (at 72 h). In the supernatant-treated group, the concentration of sucrose was decreased to 3.7 ± 0.01 mg/mL at 72 h. The sucrose decomposition was inhibited 12.5% by the treatment of *L. pentosus* MJM60383 supernatant.

### Effect of L. pentosus MJM60383 supernatant on the biofilm-associated gene expression of S. mutans

**Figure 5.**
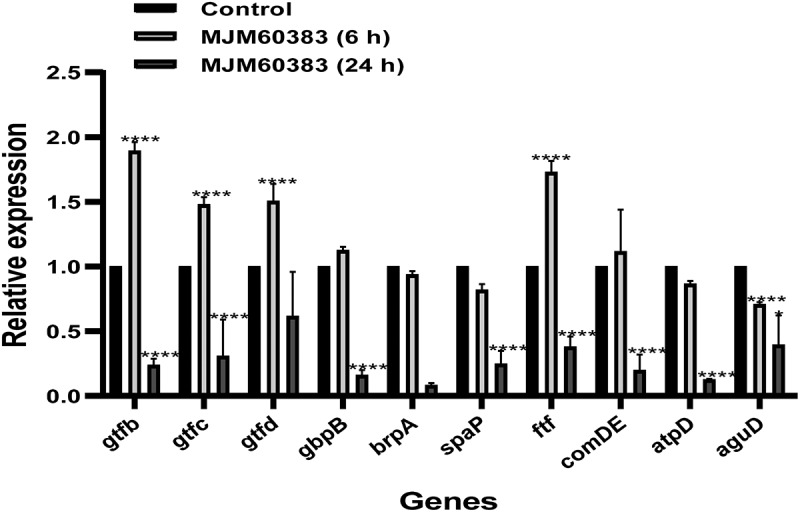
**Effect of** ***l. pentosus*** **MJM60383 supernatant on biofilm-associated gene expression**. The data are expressed as the means and SDs of three biological experiments performed in triplicate. Statistically significant differences between the presence of the supernatant-treated group and control group as analyzed using the one-way ANOVA with Dunnett’s multiple comparison test. *p <0.05, ****p <0.0001.

To explore the mechanisms of the inhibitory effect of *L. pentosus* MJM60383 supernatant, we evaluated the expression level of biofilm-related genes at 6 (initial attachment phase) and 24 h (plateau-accumulated phase) after treatment with the *L. pentosus* MJM60383 supernatant. As shown in Figure 5, genes *gtfb*, *gtfc*, *gtfd*, and *ftf* were significantly upregulated at 6 h compared with the control group. However, most of the genes, except for the *aguD* gene, were significantly down-regulated by treatment with the supernatant at 24 h. These results showed that *L. pentosus* MJM60383 only can inhibit biofilm-related gene expression at the plateau accumulated phase, rather than the attachment phase.

## Discussion

Probiotics have been widely studied to prevent and treat diseases that are associated with gut microbiota. Although the oral cavity harbors the most abundant microbes in humans, studies on oral probiotics are limited. Recently, some lactic acid bacteria, like *Lactobacillus* [31], *Streptococcus* [32], and *Weissella* [33] are reported to benefit oral health, but still more studies are needed in this important field. This study aimed to evaluate the oral probiotic potential of *L. pentosus* MJM60383, which was isolated from fermented vegetables, and the anti-cariogenic effect of *L. pentosus* MJM60383 against *S. mutans* was investigated. LGG has been reported to have good effects on children’s oral cavities by reducing the risk of dental caries and the number of *S. mutans* [34]. Thus, LGG was used as a control strain in this study.

First of all, a probiotic should be safe and can survive in the human body when introduced. In this study, the oral probiotic properties were characterized by safety assessment (biogenic amine production, hemolytic activity, antibiotics susceptibility), and survival in the simulated oral environment (tolerance to lysozyme, attachment to oral epithelial cell). Strain *L. pentosus* MJM60383 has no hemolytic activity, no biogenic amine production, and no D-lactic acid production, which is safe for consumption. Antibiotic susceptibility is one of the important safety requirements for potential probiotic strains that they should not carry transferable antibiotic resistance genes [35]. The spread of these genes among bacterial species may lead to enhanced antibiotic resistance [36]. Therefore, potential oral probiotics must test their antibiotic susceptibility. In our study, *L. pentosus* MJM60383 showed susceptibility to chloramphenicol, ampicillin, gentamycin, streptomycin, erythromycin, and clindamycin, but was resistant to vancomycin, tetracycline, and kanamycin. Resistance to vancomycin by the lactic acid bacteria was common and was not required according to the guideline of EFSA1 [8]. It was reported that the vancomycin-resistant gene of LGG is located in the chromosome, not in the plasmid, and the vancomycin-resistant gene is not related to the enterococcal van genes [37]. In addition, the genetic basis of resistance for tetracycline and kanamycin needs further exploration according to the EFSA guidance [18] (EFSA, 2012).

*L. pentosus* MJM60383 showed higher antagonistic activity against *S. mutans* than LGG. Generally, LAB was reported to inhibit pathogens by producing organic acid, hydrogen peroxide, and bacteriocins. Thus, the production of organic acids and hydrogen peroxide by *L. pentosus* MJM60383 was investigated. *L. pentosus* MJM60383 produced H_2_O_2_ (0.06–0.14 mM), which was higher than LGG (<0.015 mM). *L. pentosus* MJM60383 also produced lactic acid (13.89 ± 0.12 g/L), which was similar to LGG (14.05 ± 0.13 g/L). Although acid production by LAB may contribute to antimicrobial activity, excessive acid production may damage the tooth enamel and lead to tooth decay. As consumption of LGG was reported to benefit children’s dental health [38], lactic acid produced by *L. pentosus* MJM60383 (at a similar concentration of LGG) may not damage human teeth.

Second, as an oral probiotic, it should survive in the oral cavity and colonize the oral tissues. Lysozyme in the oral cavity can disrupt the cell wall and lead to some Gram-positive bacteria cell lysis. Lysozyme was found in the oral cavity at concentrations of 40–280 µg/ml, or up to 477 µg/ml in some conditions [39]. *L. pentosus* MJM60383 was able to tolerate lysozyme at the concentration of 1 mg/mL, which is 2-fold the maximum concentration of lysozyme in the oral cavity. Thus, *L. pentosus* MJM60383 has the potential to survive in the oral cavity. Oral microbes can directly attach to the oral epithelial tissue, which is covered with a thin mucosal layer composed of mucins in oral saliva [11]. Therefore, oral probiotics should colonize the oral cavity by possessing a higher adherence ability to oral epithelial tissue instead of to teeth. In the present study, *L. pentosus* MJM60383 exhibited more adhesive ability to YD-38 cells (20%) than LGG. The adhesion of lactobacilli to oral epithelial cells prevents the colonization of pathogens [40]. This result suggested that strain *L. pentosus* MJM60383, although isolated from fermented vegetables, has the potential to colonize the oral cavity. Some lactobacilli have been reported to possess S-layer proteins that can enhance their adherence to epithelial HT-29 cells and Caco-2 cells from the colon [41]. Thus, *L. pentosus* MJM60383 may adhere to the oral epithelial cells via S-layer protein.

Cell aggregation involves the interaction of cell surface components, such as soluble proteins, carbohydrates, and lipoteichoic acid [19]. *L. penosus* MJM60383 showed good co-aggregation ability with *S. mutans*. The co-aggregation of *L. penosus* MJM60383 with *S. mutans* reduced the opportunity of *S. mutans* auto-aggregation and adherence to the tooth surface, thus may partially reduce the risk of dental caries.

Another important trait of oral probiotics is anti-biofilm activity. *S. mutans* can produce biofilm which can protect them from the harsh environment and survive in the oral cavity. Thus, inhibition of biofilm can enhance the beneficial effect of *L. pentosus* MJM60383 in the oral cavity. In this study, biofilm formation of *S. mutans* was inhibited either by *L. pentosus* MJM60383 culture (co-culture model) or supernatant treatment. The supernatant treatment showed stronger activity than the co-culture model. In the co-culture model, only a few bacteria were inoculated into the BHI media, the main mechanism for the inhibition of biofilm may attribute to cell–cell interactions, like co-aggregation and auto-aggregation. Furthermore, in the co-culture model, *L. pentosus* MJM60383 was cultured in BHI broth in which lactic acid bacteria may not secrete as many metabolites as in MRS broth. Generally, the growth and metabolite production of *Lactobacillus* was influenced by the culture conditions, like media, pH, and temperature. Yang, E. *et al.* reported that bacteriocin production was significantly different in MRS versus BHI medium [42]. The active compounds produced by *L. pentosus* MJM60383 in the MRS medium may contribute strongly to the inhibition of biofilm by the supernatant.

Due to the strong activity shown by the supernatant, the mechanism study was mainly done in the supernatant-treated model. *Lactobacillus* can produce bioactive compounds that suppress the biofilm development of pathogens. Until now, only a few studies reported the effect of *Lactobacillus* supernatant on *S. mutans* biofilms. In this study, *L. pentosus* MJM60383 supernatant significantly inhibited *S. mutans* biofilm formation even treated with catalase, trypsin, or neutralized. These results suggested that hydrogen peroxide, lactic acid, or bacteriocin contributed to the anti-biofilm activity of *L. pentosus* MJM60383 supernatant, but they were not the only reason for the activity. Wasfi R *et al.* [43] reported that biosurfactant produced by *L. acidophilus* can inhibit the biofilm formation of *S. mutans*. Gu, *et al.* [44] discovered novel iminosugar compounds that can interfere with the biofilm formation of *S. mutans*. It is necessary to explore the active compound in further study.

Insoluble glucan synthesized by *S. mutans* is the main component of biofilm and can enhance pathogenicity by mediating bacterial adhesion to tooth surface [45]. Our study showed that *L. pentosus* MJM60383 supernatant significantly inhibited the production of water-insoluble EPS by *S. mutans* (Figure 3C). Furthermore, *L. pentosus* MJM60383 supernatant remarkably inhibited the adhesion of *S. mutans* (Figure 3B). Therefore, the reduction of insoluble glucan may also contribute to the anti-adherence activity of the supernatant.

*S. mutans* can secrete glucansucrase (also known as glucosyltransferase), an extracellular enzyme, to split sucrose and utilize the resulting glucose molecules to build exopolysaccharide, thereby contributing to the pathogenesis of dental caries [46]. We found that sucrose decomposition was reduced by the treatment of *L. pentosus* MJM60383 supernatant throughout the time course. This result suggested that the *L. pentosus* MJM60383 supernatant may inhibit the enzyme activities of glucansucrases or their expression. Ahn, Ki Bum *et al.* reported [26] that lipoteichoic acid of *L. plantarum* KCTC10887BP, a cell-wall component of gram-positive bacteria, reduced the biofilm formation of *S. mutans* by interfering with sucrose decomposition and resulted in the reduction of exopolysaccharide synthesis. However, to the best of our knowledge, this is the first report that *Lactobacillus* culture supernatant decreased *S. mutans* biofilm formation by inhibiting sucrose decomposition.

Glucosyltransferase enzymes encoded by *gtf* genes are one of the important virulence factors, which can synthesize glucan to form biofilm in *S. mutans*. Among *gtf* genes, gene *gtfb* is responsible for the synthesis of insoluble glucans, *gtfc* is involved in synthesizing both insoluble and soluble glucans, and *gtfd* mainly synthesizes soluble glucans [47]. Gene *gtfb* and *gtfc* have also been identified to be related to bacterial adherence and the stable structure of biofilm [48,49]. In this study, the expression of *gtfb*, *gtfc, gtfd*, and *ftf* was significantly reduced by the treatment with *L. pentosus* MJM60383 at 24 h. The decrease of *gtfb* and *gtfc* expression could demonstrate the anti-biofilm effect of *L. pentosus* MJM60383 supernatant by inhibiting the adherence of *S. mutans* to the surface and EPS synthesis. Thus, *L. pentosus* MJM60383 may restore the oral microenvironment by inhibiting the colonization of *S. mutans* to the oral cavity [50]. Although the *gtfb*, *gtfc*, *gtfd*, and *ftf* genes were upregulated at 6 h, sucrose decomposition was inhibited. This result indicated that the supernatant may directly inhibit the enzymatic activity of glycosyltransferase at the initial stage.

In addition, some other virulence genes involved in bacterial adhesion, biofilm signal transduction, and acid tolerance were affected by the treatment with *L. pentosus* MJM60383 supernatant. Glucan-binding protein (GBP) encoded by gene *gbp* can adhere to glucan and mediate oral bacterial aggregation, thereby promoting dental plaque formation [51]. *S. mutans* produces SpaP protein that contributes to the initial adherence and resulted in the formation of the bacterial community [52]. The gene *comD* encodes a histidine kinase receptor (ComD) and the gene *comE* gene encodes a cognate response regulator of the competence-stimulating peptide (ComE) in *S. mutans. ComD* and ComE are involved in a two-component signal transduction system, a quorum-sensing cascade of *S. mutans* [1]. The gene *brpA* encodes a predicted surface-associated protein which is known to regulate biofilm formation [53]. *S. mutans* can produce organic acid and endure acid in the enclosed biofilm environment, which results in demineralization [54]. The protein *atpD* and *aguD* produced by *S. mutans* enable *S. mutans* to conduct metabolic processes under acidic circumstances, resulting in acid tolerance [55]. The reduction of gene expression of *atpD*, *and aguD* in *S. mutans* can reduce its acid tolerance, survival, and bacteriostasis [56]. In our study, *L. pentosus* MJM60383 supernatant could not alter the expression of these genes (*BrpA*, *ComDE, SpaP, Gbp, aguD, atpD*) at 6 h. However, the expressions of tested genes were dramatically decreased after treatment with *L. pentosus* MJM60383 supernatant for 24 h. This result showed that the anti-biofilm activity of *L. pentosus* MJM60383 supernatant might mainly downregulate the expression of genes in the active accumulated phase (6–12 h) and initial plateau accumulated phase (12–20 h) [57].

In summary, this study demonstrated that *L. pentosus* MJM60383 showed oral probiotic properties and anti-biofilm activity against *S. mutans*. The possible anti-biofilm mechanism could be attributed to the inhibition of sucrose decomposition through inhibiting glucansucrases enzymes by *L. pentosus* MJM60383 supernatant. Thus, *L. pentosus* MJM60383 can be used to develop oral probiotics. In future work, *in vivo* or clinic studies should be performed to confirm the activity of *L. pentosus* MJM60383 as an oral probiotic.
